# Research on the child healthcare system in China from the perspective of equal health benefits: Beijing as the case study

**DOI:** 10.1111/cch.12953

**Published:** 2022-01-11

**Authors:** Qi Zhuang, Yunzhi Peng, Jieyu Zhou, Peter Coyte

**Affiliations:** ^1^ School of Management University of Chinese Academy of Social Sciences Beijing China; ^2^ Central University of Finance and Economics, Administrative Management Beijing China; ^3^ Business School University of Edinburgh Edinburgh UK; ^4^ School of Public Health University of Toronto Toronto Canada

**Keywords:** child healthcare, children's preventive healthcare, equal health benefits, referral system

## Abstract

**Background:**

With government's proposal of the Healthy China Strategy, universal health has become the focus of increasing attention in China. As a special group, children's health is the basis and start of the national health. The construction of children's healthcare system should be given the priority. This study applied the concept of health equal benefits and conducted a systematic and in‐depth theoretical and empirical research on the children's healthcare system in Beijing, aiming at proposing relevant countermeasures and suggestions such as constructing the path of children's health care system in China.

**Methods:**

This study adopted the way of questionnaire on children's basic information, health status, health service demand, insured situation and system satisfaction and then analysed the children's health, healthcare, satisfaction influence factors and their healthcare needs based on a survey in Beijing China. Methods such as descriptive statistics, analysis of variance and logistic regression are adopted to analyse the correlation between factors affecting children's health.

**Results:**

The findings show that overall health of the children in Beijing was better. However, there are still problems such as insufficient resource coverage and incorrect cognition in some groups. Their mental health and support need to be put emphasis on. The popularization of medical insurance policies for urban and rural residents in Beijing needs to be further improved. The burden of seeing a doctor is slightly lower than before, and the contribution to protecting children's health is not strong enough.

**Conclusions:**

Through the survey of children in Beijing, it can be seen that there are still some problems existing in the healthcare system of children in China from the perspective of equal benefit. This study draws policy attention to the issues of financing, service contents, targets, service level and the degree of social recognition.

**Key messages**

- The construction of children's healthcare system in China needs to be given the priority.
- This study applied the concept of health equal benefits and conducted a systematic and in‐depth theoretical and empirical research on the children's healthcare system in Beijing.
- The findings show that overall health of the children in Beijing was better.
- However, there are still problems such as insufficient resource coverage and incorrect cognition in some groups.
- This study draws policy attention to the issues of financing, service contents, targets, service level and the degree of social recognition.

## 1 INTRODUCTION

Healthy China Strategy has emphasized the importance of healthcare to the national strategic level to achieve the transformation from medical insurance system to healthcare systems (Xi, 2017). Children are the future of the country and the nation, and their healthy growth is also related to the stability and happiness of every family. According to data from the official website of the National Bureau of Statistics of China, as of 2018, the population of 0–14 years old was nearly 230 million (The National Bureau of Statistics of the People's Republic of China, 2018). This means that the huge group of children should be given enough attention in the construction of the healthcare system. At the same time, children, as a special group, have the characteristics of high morbidity and high outpatient rate. Because of these special characteristics, children under the same system cannot enjoy the same level of protection as other types of groups. Therefore, from the perspective of equal benefits, a healthcare system for children should be established, and the characteristics of the mechanism should be fully utilized to enable children to grow up healthily, which in turn is conducive to national development and social progress.

Equal benefits can be understood as the equalization state in the field of basic public services. There is a causal relationship between these. Equal benefits are the final effect to be achieved, and the equalization of basic public services is the way and actual performance to achieve equal benefit. At the present stage, the equalization of basic public services in China mainly includes three levels of meaning. The first level is that the central or local government should make clear provisions on the basic public services and standards in the area under its jurisdiction; the second level is that all residents should have the financial capacity to pay for basic public services and the central government (or the local government) must ensure that they do; the third level is that all residents should have the right and access to basic service facilities, irrespective of whether they are in urban or rural or regional areas (Ding, 2012).

Specific to the equal benefit in the field of children's healthcare, it mainly includes two aspects. First of all, as a special group in healthcare system, children should enjoy the same healthcare rights as other groups. Secondly, within the group, it is necessary to ensure that every child has the opportunity and the right to get the equal healthcare services. This requires the improvement of the corresponding medical service delivery system. Ensuring that children's healthcare resources are properly allocated at different levels of the healthcare delivery system will reduce the cost and enable more children to consume qualified healthcare services. The key to ensure that this system really works is the hierarchical diagnosis and treatment system and the corresponding incentive and restraint mechanism (Zhai & Xu, 2013). Among them, hierarchical diagnosis and treatment means that every child patient must first go to the specified Level I medical institution. If it cannot provide needed treatment, the doctor will issue a referral form allowing the child to be referred to the Level II medical institution, and the individual patient has no right to go beyond the Level II medical institution. In addition, when the patient's condition is alleviated, the patient needs to be transferred from a higher level to a lower level medical institution, namely, the mutual referral system. Hierarchical diagnosis and treatments distinguish the functions of medical institutions, promote the coordination and distribution of responsibilities among institutions, avoid disorderly and vicious competition and make the most effective use of medical resources. However, this grading system is an idealized design, and it is easily affected by information asymmetry, induced consumption and other phenomena in reality. Therefore, to make this system structure work in a healthy manner in reality, it is necessary to establish a supporting incentive and restraint mechanism.

This study applied the concept of health equal benefits and adopted the analytical framework of institutional analysis and mechanism design to conduct a systematic and in‐depth theoretical and empirical research on the children's healthcare system based on a survey in Beijing, aiming at finding out problems and proposing relevant countermeasures and suggestions for the existing children's healthcare system in China.

## 2 CHILD HEALTHCARE POLICIES IN BEIJING

In the implementation of the Healthy Beijing 2030 Plan of Action (2018–2020), Beijing government makes it clear that it should improve national healthcare system, which specifically includes improving health service system, medical insurance system and the pharmaceutical supply system and so on. In terms of optimizing child health services, it is mainly divided into infant and young child health services and adolescent health services.^1^ Among them, health services for infants and young children include regular review of baby‐friendly hospitals, improvement of the early childhood comprehensive development service referral network, training of healthcare personnel in kindergartens, strengthening health management of premature babies, health services for adolescents include establishing and improving youth health service system, a joint supervision system for education and health and a student disease prevention and control model linked by families, schools and health institutions.

In terms of health education, the Health Month Activity themed ‘Home and school work together to love and protect eyes’ was carried out in Beijing, the work of ‘Experts into the campus for health lectures’ was continued, and a series of health education activities in primary and secondary schools were carried out by instructing parents and students to conduct health management^2^ so as to ensure children's physical and mental health growth.

In terms of promoting the service of family doctors for children, Beijing has a total of 6378 family doctors and 4100 general practitioner teams. More than 7.4 million residents have signed up family doctors, with a total signing rate of 35% in 2019.^3^ Beijing Health and Family Planning Commission signed on Further Strengthening the Family Doctor Service Announcement concerning the work of more emphasis on priority for key group's signing service,^4^ and the personalized service package would be based on the demand of children aged 0–6, mainly divided into the basic medical services, public health and health management services. The latter will be more targeted, including home visits, the first‐month health management (within institutions) of newborn babies, infants and young children health management (0–3 years old), preschool children health management (4–6 years old), health problems, vaccination and 0‐ to 3‐year‐old children health management services of traditional Chinese medicine, health consulting service, information service, the content of health education and other services.

Beijing Municipal Government has implemented the Student and Children's Seriously Ill Insurance, which is part of ‘One Elderly and One Young’ Medical Insurance system. It covered Beijing non‐agricultural household and registered students in various schools divided by the administrative area, as well as children who are not in school, whereas those registered students with Beijing agricultural household registration a voluntarily choose to whether participate in this insurance. In 2007, each insured child paid RMB 100 of insurance premium per school year. In 2014, it was adjusted to RMB 160 per year, in 2017 to RMB 180 per year, in 2019 to RM B300 per year and in 2021 to RMB 325 per year.

The Measures on Basic Medical Insurance for Urban and Rural Residents of Beijing came into effect on 1 January 2018. In 2021, the reimbursement standard has been adjusted again, and the reimbursement ratio has been further refined, which is divided into outpatient and inpatient parts.

1. Outpatient reimbursement: The limit line for outpatient and emergency reimbursement in 2021 has been raised by RMB 500–RMB 4500. The starting line for medical institutions of the Level I and below is RMB 100/year, and 55% of the medical expenses within the scope of medical insurance can be reimbursed. The starting line for Level II and III medical institutions is RMB 550/year, and the medical expenses within the scope of medical insurance can be reimbursed by 50%.
2. Reimbursement of hospitalization: The limit line of reimbursement is RMB 250 000. The starting line of the Level I and below medical institutions is RMB 300/year, the medical expenses within the scope of medical insurance can be reimbursed by 80%; For Level II medical institutions, it is RMB 1300/year, and the medical expenses within the scope of medical insurance can be reimbursed by 78%. For Level III medical institutions, it is RMB 1300/year, and the medical expenses within the scope of medical insurance can be reimbursed by 75%–78%.^5^

From ‘One Elderly One Young’ Policy to the transition of the Urban and Rural Residents' Basic Medical Insurance, this means that the construction of Beijing medical care system has been improved and demonstrated as multilevelled, which can be seen as gradually achieve the goal of ‘everyone will have access to medical insurance’ and at the same time also means that the more emphasis has been put on this special group of children increasingly and more rights and interests of the children have been effectively guaranteed. But medical care and healthcare of children are two different concepts that have shown up the transition from ‘cure disease’ to ‘health’. So at this stage, there are still some problems in the children's health service supply in Beijing. The construction of the child healthcare system also needs to be gradually realized under the background of a sound universal healthcare system.

## 3 METHODS

### 3.1 Participants

In the process of the survey, the project team adopted the way of questionnaire on children's basic information, health status, health service demand, insured situation and system satisfaction and then analysed the children's health, healthcare services, satisfaction influence factors and their healthcare needs. Stratified sampling method was taken according to 16 administrative regions in Beijing. The objects of study are the children of 0–18 age group in Beijing and selected the children's parents, grandparents or other major guardians as targeted questionnaire respondents. Their willingness to participate in this survey was respected. The overall number was 5620, and this population is stratified by administrative region, and random sampling is carried out according to the ratio of sample size to the total number of 1:5. A total of 1124 questionnaires were selected in this survey, among which 1079 were valid, with an effective recovery rate of 96.00%.

### 3.2 Procedure

Prior to collecting information, we first obtained the consents of the parents, grandparents or other major guardians. Online questionnaires were distributed to them. Respondents voluntarily completed the questionnaires. During the survey, the researcher and the team remained in contact, answered questions and mentored the participants' activity.

### 3.3 Instruments

#### 3.3.1 Basic information

In this part, we made statistics on children's gender, age, household registration (health insurance system was based on this policy), average monthly household income and total monthly household expenditure. In the next part, we would use these statistics to evaluate the degree of equity and correlation between the child health and these factors.

#### 3.3.2 Child health status

In this part, we evaluated the children's physical and mental health. For physical health, using a 5‐point Likert scale (1 = *excellent*, 5 = *not good*), the child's general health was measured. And using 4‐point Likert scale (1 = *no change*, 5 = *hard to say*), changes in child's health condition was measured. Physical examination and vaccination situation were also measured in this part.

For mental health, we focused on the general psychological condition using a 5‐point Likert scale (1 = *very happy*, 5 = *very unhappy*). In the meantime, we inquired the conditions of child's friendship and emotional support and guidance, as well as their after‐school activities.

#### 3.3.3 Medical insurance status

In this part, we put emphasis on the participation and satisfaction with the medical insurance. For the satisfaction, we used a 5‐point Likert scale (1 = *very satisfied*, 5 = *very dissatisfied*).

#### 3.3.4 Data analysis

In this paper, SPSS software is used to analyse the data obtained from the questionnaire survey. Methods such as descriptive statistics, analysis of variance and logistic regression are adopted to analyse the correlation between factors affecting children's health.

## 4 RESULTS

### 4.1 Descriptive statistical analysis

#### 4.1.1 Children's health status

##### Children's physical health

As it shown in the Table 1, this indicated that the overall health of the children was better and the children's health is in a good trend in the near future.

**TABLE 1:** Overall children's health statistics

Overall situation	Options	Frequency	Percentage (%)
Child's general health	Excellent	362	33.5
	Very good	412	38.2
	Good	227	21.0
	Average	75	7.0
	Not good	3	0.3
Changes in the child's condition	No change	497	46.1
	Better	378	35.0
	Worse	71	6.6
	Hard to say	133	12.3

Regular physical examinations account for the vast majority (86.9%), and the most important reason for not allowing children to participate in regular physical examinations is that communities and schools do not provide corresponding services. Therefore, focus should be put on strengthening the coverage of children's physical examinations in communities and schools and at the same time do more publicity work to let more parents know about these services, so as to further increase the coverage of children's regular physical examinations, meet the needs of children's health and improve child health protection.

Among the vaccine‐related variables, a total of 997 responses asked children to be vaccinated on a regular basis, accounting for 92.4%, indicating that the work has been effective. By frequency analysis, it can be seen that the overall safety of domestic vaccine is accepted by the public, although there are a small number of groups doubt about it. What's more, there are still some groups that have not yet clearly recognized the need for vaccines for children, and therefore, the popularization of science should be strengthened, and people's concept of health should be cultivated. At the meantime, emphasis should also be placed on ensuring the quality of vaccines to reassure parents.

##### Children's mental health

The children interviewed are generally in good psychological condition. But there are still nearly 10% of children living without friends to share, which means that these children are more likely to live alone and thus need to pay more attention to their psychological changes. Reasonable mental health education is particularly critical.

And it also indicated that most parents attached great importance to the child's emotional and mental health and gave timely guidance. However, at the same time, there are still a very small number of parents that neglected to guide their children's emotions for some reasons, which should also play a corresponding role in such children's school and society so as to create a good psychological growth environment for children and ensure their mental health.

From the results of children's after‐school activities' descriptive statistics, it can be seen that ‘Doing homework’ is the item with the highest proportion, with a frequency of 825, accounting for 76.5%. And the extracurricular activities of children in Beijing are relatively undiversified, with homework and extracurricular reading taking up a much higher proportion than others and Internet‐related options taking up only a small proportion. Parents should give their children more free time to do what they are interested in.

#### 4.1.2 Medical insurance status

##### Participation in medical insurance

There are still 20% of the respondents who did not participate or did not understand the insurance policy. This shows that the popularization of medical insurance policies needs to be further improved.

There are a total of 494 cases of children with non‐Beijing registration. Among them, 278 cases have participated in the medical insurance for urban and rural residents in their household registration, slightly more than 216 cases who have not participated in.

As for the sorts of medical insurances for children, generally speaking, the insurance status is good. More than 95% of the children have medical insurance, which provides a good protection for children's health. In addition, more than half of the respondents have purchased commercial insurance for their children, showing that they attach great importance to children's health, which is conducive to better implementation of children's health protection.

##### Analysis of satisfaction with insurance participation

Nearly 70% of the respondents thought that the procedures were convenient or very convenient, which shows that the convenience of procedures is generally accepted by the masses.

Among these 381 respondents who have experienced reimbursement, the convenience of reimbursement is satisfactory, but there is still room for improvement.

In the question of whether the burden of seeing a doctor is reduced or heavier compared with not being insured, it can be seen that the burden of seeing a doctor is slightly lower than before and the contribution to protecting children's health is not strong enough. The effect of easing the burden is not obvious enough.

In the variable of ‘satisfaction with the Basic Insurance’, the statistics indicated that respondents were generally satisfied with the Basic Insurance.

### 4.2 Correlation analysis of factors affecting children's health in Beijing

#### 4.2.1 Correlation analysis of health, income and expenditure

The income variable was ‘average monthly household income’, the expenditure variable was ‘total monthly household expenditure’, and the health variable was ‘overall physical health status of the child’, ‘overall mental health status of the child’ and ‘whether the child has had any illness in the past 6 months’. Because the variables are classified and ordered variables, the Spearman correlation coefficient is used for analysis. The respective Spear correlation coefficients are listed in the table, with *P*‐values in brackets.

As can be seen from the correlation analysis in Table 2, just ‘average monthly income’ and ‘whether the child has the illness within last six months’ were a significant negative correlation. The *P*‐value was 0.001, which was significant at the 1% significance level, indicating that the probability of the children in families with high monthly average income in the past 6 months was significantly lower than that of families with low monthly income.

**TABLE 2:** Analysis of the correlation between health, income and expenditure

	Average monthly income	Average monthly expenditure
Physical health	0.015 (0.621)	−0.031 (0.302)
Mental health	−0.011 (0.706)	−0.042 (0.171)
Whether the child has the illness within last 6 months	−0.131^**^(0.001)	−0.045 (0.144)

^*^ *P* < 0.5.

^**^ *P* < 0.01.

None of the other variables passed the 5% significance test, indicating that the average monthly household income and monthly average expenditure are not statistically significant related to the child's physical and mental health. Children from families with higher income and expenditure are not better than children from families with lower expenditures are healthier, and there is no significant correlation between the average monthly expenditure and whether they are ill within 6 months.

The Spearman correlation coefficient and *P*‐value of children's physical health and mental health were 0.496 and 0.001, respectively, which were significantly positively correlated at the 1% significance level, and the degree of correlation was very high, indicating that parents who believed that their children were physically healthy were also significantly inclined to believe that their children were mentally healthy.

#### 4.2.2 Correlation analysis of Basic Medical Insurance satisfaction and economic situation

For the degree of satisfaction with the Basic Medical Insurance, the variable ‘How satisfied are you with the Basic Medical Insurance in general’ was selected, and for economic situation, 10 variables were selected as follows: household registration, gender, relationship with the child, age of the child, education level, marital status, employment status, type of employment, the average monthly total household income and total monthly household expenditure.

Because the degree of satisfaction is an ordinal variable and the living economic situation is a categorical variable, a chi‐square test is conducted on the household registration, gender, relationship with children, marital status, employment status and employment category variables, and Pearson chi‐square value and *P*‐value in brackets are listed. Spearman's correlation coefficient is selected for correlation analysis of children's age, education level, average monthly household income and total monthly household expenditure. The respective Spear correlation coefficients are listed in the table, with *P*‐values in brackets. The results are organized as follows.

It can be seen from Table 3 that the three variables of household registration, gender and employment status passed the chi‐square test at the 1% significance level, indicating that there are significant differences in satisfactions among Beijing household registration and non‐Beijing household registration, genders and employment conditions for Basic Medical Insurance. The relationship with children was significant at the 5% significance level, indicating that there were also significant differences among different relationships with children, that is, parents, grandparents, siblings, etc., have different satisfaction level. Further analysis was done to obtain the correlation coefficient between the satisfaction level and genders. The correlation coefficient was 0.069, and *P* was 0.024 and at the 5% significant level showed weak positive correlation level of significance. Because the satisfaction variable assignment from 1 to 5 is gradually diminishing, the gender variable 1 for male and 2 for the female, the positive coefficient indicated that male satisfaction was significantly higher than female. There was no significant difference in satisfaction among respondents with different marital status and different employment categories.

**TABLE 3:** Chi‐square test of satisfaction degree and life economic situation

	Satisfaction level
Household registration	32.682^**^ (0.001)
Gender	15.446^**^ (0.004)
Relationship with children	25.229^*^ (0.014)
Marital status	2.782 (0.595)
Employment status	49.150^**^ (0.002)
Employment unit	38.210 (0.551)

^*^ *P* < 0.5.

^**^ *P* < 0.01.

It can be seen from Table 4 that none of the four variables passed the Spearman correlation analysis at the 5% significance level. Therefore, there was no statistically significant correlation between children's age, education level, average monthly family income, total monthly family expenditure and satisfaction.

**TABLE 4:** Correlation analysis between satisfaction degree and economic situation

	Satisfaction level
Child age	0.018 (0.555)
Education level	0.004 (0.900)
Average monthly household income	0.056 (0.074)
Total monthly household expenditure	0.043 (0.171)

^*^ *P* < 0.5.

^**^ *P* < 0.01.

## 5 DISCUSSION

Through the above analysis on present situation of children's health in Beijing, it shows there are still some problems in children's health services, following mainly from the view of financing mechanism, service content, service level and degrees of social acceptance.

### 5.1 The financing operation mechanism remains to be improved

The financing mechanism of the current Basic Medical Insurance System for Urban and Rural Residents (BMISUR) has been greatly improved compared with before, and the level of financial subsidies has been continuously increased, reducing the pressure on residents to seek medical treatment, but the overall financing mechanism still needs to be improved. First of all, in terms of financing structure, the government, society and individuals bear an unreasonable proportion of the responsibility. The current BMISUR in Beijing implements a combination of individual payment and government subsidies and encourages collectives, employer or other social and economic organizations to provide support or subsidies. In 2021, the per capita funding standard for students and children is RMB 1970 per year, including RMB 325 per year for individual contributions and RMB 1645 per year for financial subsidies (Xicai Net, 2020). It can be seen that the current financing structure has not been truly diversified and is still dominated by government financial subsidies and individual contributions. The social support is limited and cannot be used as a sustainable and stable source of financing. Secondly, in terms of financing methods, the principles of voluntary participation and centralized payment collection affect the stability of funding. It can also cause children in poor family uninsured. At the same time, this voluntary principle will make it difficult to truly achieve the basic goal of the ‘universal coverage’. Finally, in the financing standard, the contribution standard is single immobilized. The current individual payment standard for children is fixed at RMB 325. However, the medical expenses of children with different diseases are also varied, and the payment according to the same standard cannot meet different needs and does not reflect the idea of equal benefits.

### 5.2 The particularity of this group is not prominent

In the Action Plan for improving the national healthcare system in Beijing, no specific policy measures have been put forward for children. In terms of the scope of medical reimbursement, it mainly focuses on disease treatment, whereas the contents of reimbursement for prevention and healthcare are not enough. As a special vulnerable group, children, especially in the newborn stage of infant health examination, health immunization and other projects are necessary for children, and the limitation of the scope of medical reimbursement makes children lose the opportunity to enjoy the treatment of medical reimbursement. From this perspective, it is also contrary to the concept of equal benefit. Therefore, the children's health service supply in Beijing lacks sufficient pertinence.

### 5.3 Service contents needs to be expanded and enriched

In the context of the Healthy China Strategy, the healthcare system includes not only physical health but also mental health, and the latter is particularly important. However, the analysis shows that children enjoy fewer mental health services and the service providers fail to realize the importance of mental health training for children.

### 5.4 Service level needs to be improved and factors that influence the willingness to participate the BMISUR need to be considered

In the survey, as for the procedures of Beijing BMISUR, nearly 5% of people think that the participation procedures are not convenient, whereas there are some people who think the reimbursement process is inconvenient, reflecting that the administration needs to be improved. And this experience will influence the willingness to participate the BMISUR. Especially for some elderly people, they are not familiar with online payment and other online procedures. In China, lots of the children are cared by grandparents. How to provide them convenient services is a realistic problem. As for the hospital services, respondents complained for long waiting time and short diagnosis time with unsatisfied treatment. And most of the first choice of responds is going to Level III directly instead of going to the Level I institutions. This indicates that there is great room for improvement in the referral system, allocation of medical resources and professional talents in hospitals and certain measures should be taken to improve the service level so that children can enjoy better health services. According to the survey, the child's age, mother's education level, the child's household registration, whether the child is covered by health insurance other than BMISUR, the area of residence and whether the child has been sick in the last 6 months are factors that influence whether the parents purchase BMISUR for the child. BMISUR is a voluntary‐based insurance, and these influencing factors are key to expanding insurance coverage.

## 6 CONCLUSION

Child health is the foundation of the national health. Through the survey of children in Beijing, it can be seen that there are still some problems existing in the healthcare system of children in China from the perspective of equal benefit. The issues of financing, service contents, service level and the degree of social recognition need to be emphasized in the future. And corresponding measures need to be taken. First, the government should take on the responsibility of strengthen legal, regulatory and policy guarantees to protect children's health rights. Second, the financing channels should be more diversified and attach importance to the power of market. Third, the reimbursement scope and contents need to be broaden, especially for the children's preventive healthcare. Fourth, health education and mental health of children need to be emphasized besides medical care. Fifth, encouragement and regulatory measures need to be taken to strengthen the referral system and increase the investment on general practitioners and primary health institutions.

## CONFLICT OF INTERESTS

The authors declared no potential conflicts of interest with respect to the research, authorship and/or publication of this article.

## AUTHOR CONTRIBUTIONS

Qi Zhuang conceived of the study and wrote the manuscript. Yunzhi Peng helped to draft and Peter Coyte provided a variety of comments. Jieyu Zhou performed the statistical analysis. Qi Zhuang, Yunzhi Peng, Jieyu Zhou and Peter Coyte participated in its design and coordination. All authors read and approved the final manuscript.

## Data Availability

The data that support the findings of this study are available from the corresponding author upon reasonable request.

## ENDNOTES

^1^
The Beijing Board of Health (2018), Beijing implementing ‘Healthy Beijing 2030’ Action Plan (2018–2020). http://wjw.beijing.gov.cn/zwgk_20040/ghjh1/201912/t20191216_1242779.html (accessed 12 October 2020).

^2^
The Beijing Municipal People's Government (2019), The Notice from Beijing Education Committee for health education activities in Primary and Secondary Schools in 2019. http://jw.beijing.gov.cn/xxgk/zfxxgkml/zfgkzcwj/zwgzdt/202001/t20200107_1564954.html (accessed 17 July 2019).

^3^
Beijing Daily, the People's Daily (2019), The Beijing 7.4 million residents signed the family doctor. http://society.people.com.cn/n1/2019/0520/c1008-31093358.html (accessed 12 October 2020).

^4^
The Beijing Municipal Commission of Health (2019). Beijing Health and Family Planning Commission signed on further strengthening the family doctor service announcement. http://wjw.beijing.gov.cn/zwgk_20040/ztzl/fjzl/201912/t20191217_1244323.html (accessed 12 October 2020).

^5^
Xicai Net (2020), Introduction on the Reimbursement Standard of 2021 Beijing Residents of Urban and Rural Medical Insurance. The limit line of hospitalization expenses reimbursement is RMB 250 000. https://www.csai.cn/baoxian/1318336.html (accessed 12 October 2020).
